# Efficacy, Satisfaction, and Compliance: Insights from 15 Years of Botulinum Toxin Use for Female Urgency Urinary Incontinence

**DOI:** 10.3390/toxins16080332

**Published:** 2024-07-26

**Authors:** Margarida Manso, João Diogo Soares, Margarida Henriques, Francisco Botelho, Carlos Silva, Francisco Cruz

**Affiliations:** 1 Urology Department, Centro Hospitalar Universitário São João, 4200-319 Porto, Portugal; 2 Faculdade de Medicina da Universidade do Porto, 4200-319 Porto, Portugal; 3 Instituto de Investigação em Ciências da Vida e Saúde, Escola de Medicina da Universidade do Minho, 4710-057 Braga, Portugal

**Keywords:** female urinary incontinence, urgency urinary incontinence, overactive bladder, bladder botulinum toxin, Botox^®^

## Abstract

Urgency urinary incontinence (UUI) refractory to medical treatment poses significant challenges despite advancements. This study evaluates the efficacy of intravesical botulinum toxin for UUI and identifies factors influencing treatment outcomes. Among 368 women receiving botulinum toxin injections, 74.5% achieved a complete discontinuation of pad usage. Predictors of efficacy included lower pre-treatment pad usage and the absence of prior sling placement. Patients often required repeat injections (60.3%), with younger age and satisfaction correlating with treatment repetition. The interval between injections averaged 18 months, influenced by logistical challenges and patient preferences. Despite concerns about diminishing efficacy, subjective perceptions did not align with objective findings. Limitations include retrospective analysis and heterogeneous clinical records. In conclusion, intravesical botulinum toxin is effective for UUI, with pre-treatment pad usage and sling placement history influencing outcomes and patient characteristics influencing treatment repetition.

## 1. Introduction

While urgency urinary incontinence (UUI) may not be the most common form of incontinence, it often prompts individuals to seek medical attention more frequently than other types. Most cases are idiopathic, and it is generally assumed to be associated with the aging process, although other risk factors are identified [1,2].

According to European Association of Urology guidelines, the first-line treatment of UUI, whether included or not in the overactive bladder (OAB) syndrome, relies on lifestyle interventions and bladder training, with second-line being pharmacotherapy. Anticholinergics have revolutionized the treatment of UUI, but the associated side effects and the need for continuous medication lead many patients to discontinue treatment. Despite valuable additions such as beta-3 agonists, a substantial number of cases either do not improve or show only limited progress. When pharmacotherapy fails or cannot be tolerated, a bladder wall injection of botulinum toxin is one of the third-line therapies [2,3].

However, it is essential to acknowledge that even with this therapy, there are both refractory patients and individuals who, over the long term, discontinue treatment despite initially positive responses. The reasons for such discontinuation remain unclear and cannot be determined by pivotal studies. Therefore, in this study, we aim to clarify if we can predict which patients will respond favorably or unfavorably, if there are factors influencing the efficacy and duration of the treatment, and which patients are more likely to request repeated injections, as well as the efficacy of botulinum toxin in treating UUI in real-life conditions.

## 2. Results

The study involved 368 women diagnosed with UUI between 2010 and the last quarter of 2023, all of whom underwent bladder injections of botulinum toxin. Patients injected after that date were not included due to the inability to properly analyze the duration of the treatment. Of the 368 patients analyzed, the mean age at diagnosis was 60.4 years (SD ± 14.1) and had an average BMI of 30.3 (SD ± 5.4). In this cohort, 27.7% had undergone a previous mid-urethral sling placement. Among those with available information, 67.7% had a transobturator tape, 25% had a single incision sling, and 7.3% had a retropubic sling.

Among the 306 patients with complete information concerning pad usage, the median number of pads used per day before botulinum toxin injection was 3 (p25–p75: 2–5), which subsequently decreased to 0 (p25–p75: 0–1) post injection. The majority (74.5%) of patients reported a complete discontinuation of pad usage after treatment, while 25.5% continued to use one or more pads. In this group are included 7.2% of patients who had no improvement.

In 60.3% of cases, additional injections were deemed necessary, with a median of 1 (p25–p75: 0–2) additional treatment (Table 1) and a median time until the second injection of 18 months (p25–p75: 11–29).

The number of pads used at baseline predicted the chance of being dry after botulinum toxin injection. As a matter of fact, it was observed that the lower the number of pads used before the treatment, the higher the likelihood of achieving a pad-free situation. Specifically, women who discontinued pads after onabotulinumtoxinA (onabotA) had an average of 3.5 pads pre-OnabotA while those who still required pads had an average of 4.8 pads pre-OnabotA (*p* < 0.05). Additionally, patients who had undergone sling placement were less likely to achieve continence. Among those without previous sling placement, 77.9% achieved pad-free status after OnabotA treatment compared with 65.8% among those with prior sling placement (*p* < 0.05). No statistically significant relationship was identified between BMI and the number of pads before and after injection.

Regarding the request for new treatments, women who repeated the injection at least twice were, on average, younger at the time of diagnosis (57.3 years, SD ± 16.1) compared to those who did not repeat injections (61.7 years, SD ± 13.1) (*p* < 0.05). Furthermore, patients who reported satisfaction were more likely to undergo repeated treatment than those who expressed dissatisfaction (*p* < 0.05).

In the multivariate analysis model, which included the variables age, BMI, previous slings, and the previous number of pads, only the previous number of pads was an independent predictor of having zero pads after injection (adjusted OR 1.12; *p* = 0.042).

The complications associated with the treatments were mild. There were 4 patients with urinary retention requiring temporary urethral catheterization (1.1%) and 29 (7.9%) with symptoms of urinary tract infection treated with oral antibiotics.

## 3. Discussion

Only onabotulinumtoxinA (Botox^®^, Allergan, Inc., Irvine, CA, USA) 100U is licensed in Europe to treat UUI/OAB, and it is relevant to note that it is more effective in curing UUI than antimuscarinics, being a third-line treatment based on the assumption that intervention therapy should follow oral medication [2,3]. Sacral nerve stimulation is also a third-line treatment; however, so far, the trials demonstrated that the neuromodulation is comparable not with 100 U but with 200 U of the same toxin. Additionally, the increasing invasiveness, more serious complications, and higher costs of the two-stage implantation procedure should be mentioned, despite the potential need for just one (two, in fact) intervention [4,5]. However, that is beyond the scope of this paper.

The introduction of intravesical botulinum toxin treatment has been a significant advancement. It began to be used to treat medical conditions by the end of the 1970s, but it was only applied in urology in 1988, being used to treat urinary incontinence a few years later [6]. The first significant studies and clinical applications of botulinum toxin for treating UUI emerged around the early 2000s, and by 2009, substantial evidence supported its efficacy for this condition, leading to broader use in clinical practice. It is a neurotoxic protein produced by *Clostridium botulinum*, with seven subtypes. Subtype A has the longest duration of action, which makes it the most clinically relevant [2,6].

Botulinum toxin subtype A consists of a heavy and a light chain linked by a disulfide bond. When it is injected, it infiltrates the nerve terminals and enters the presynaptic neuron membrane through binding of the heavy chain to the synaptic vesicle protein (SV2). The heavy chain of the toxin exhibits an affinity for polygangliosides located on the surface of neuronal terminals, thereby augmenting the toxin’s concentration on the neuronal membrane. This heightened concentration enhances the likelihood of the heavy chain encountering the protein acceptor SV2 predominantly expressed within synaptic vesicles. After toxin endocytosis, acidification within the synaptic vesicles precipitates the elimination of the disulfide bond and the dissociation of the two chains. The light chain protein, which is the true active part, is then linked to the synaptosomal nerve-associated protein 25 (SNAP-25). When the light chain links to SNAP-25, it cleaves it and inactivates it [2,6]. By cleaving SNAP-25, it hinders the proper formation of the SNARE (Soluble NSF Attachment Protein Receptor) complex. As the SNARE complex mediates the binding of neurotransmitters vesicles to the plasma membrane of the nerve terminals, so they can fuse and release acetylcholine (Ach), and, consequently, this prevents Ach exocytosis from the vesicles. Why is this Ach blockage so essential? Because if during the voiding phase it plays a crucial role in bladder contraction to facilitate urine evacuation, during the storage phase, its effect is undesirable since there is no physiological parasympathetic input to the lower urinary tract. However, ACh can be spontaneously released from nerves and non-neuronal structures, including the urothelium and suburothelium, during this phase. This release can activate afferent nerves innervating the bladder, potentially causing urgency and UUI. When the cascade of events due to botulin toxin bladder injection occurs, as Ach is not released, it cannot bind to muscarinic receptors on the bladder detrusor muscle, thereby averting undesired contractions [2,7,8]. Furthermore, adding to this motor/efferent modulation, botulinum toxin reduces the expression of sensory receptors in bladder nerves by hampering their transfer from intracellular vesicles to the neuronal membrane, also modulating the sensory/afferent component of the disease [9].

What sets different commercial forms apart is the molecular weight of the accessory protein that covers the toxin. Xeomin^®^ (Merz Pharmaceuticals GmbH, Frankfurt, Germany), Dysport^®^ (Ipsen Biopharm Ltd., Wrexham, UK), and especially Botox^®^ (Allergan, Inc., Irvine, CA, USA) are the best known. Xeomin^®^ (Merz Pharmaceuticals GmbH, Frankfurt, Germany) has a molecular weight of only 150 kDa, Dysport^®^ (Ipsen Biopharm Ltd., Wrexham, UK) around 400 kDa, and Botox^®^ (Allergan, Inc., Irvine, CA, USA) 900 kDa. Consequently, posology, the potency per weight of each brand, is unique and not interchangeable [10]. This was the reason behind the FDA introducing the non-proprietary names of incobotulinumtoxinA (incobotA) for Xeomin^®^ (Merz Pharmaceuticals GmbH, Frankfurt, Germany), abobotulinumtoxinA (abobotA) for Dysport^®^ (Ipsen Biopharm Ltd., Wrexham, UK), and, as previously mentioned, onabotulinumtoxinA (onabotA) for Botox^®^ (Allergan, Inc., Irvine, CA, USA) [2].

Despite the dose depending on the chosen toxin brand, the preparation method is similar. It is important to note that during reconstitution, vials should be gently stirred rather than shaken, as vigorous agitation may disrupt the delicate disulfide bond linking the light and heavy chains of the botulinum toxin molecule [10]. Regarding its administration, it requires the injection of a toxin solution into the bladder wall, as the toxin fails to cross the urothelium upon instillation into the bladder. Efforts to facilitate this process have so far yielded limited success or await a definitive validation of efficacy. For patients with OAB undergoing a bladder injection of onabotA, the standard administration implies 20 injections of 0.5 mL each, classically sparing the trigone. However, despite these recommendations regarding the number of injection sites, many clinicians opt for a reduced number of injection points. Non-randomized controlled studies have utilized one or three injection sites, each receiving 1–5 mL of solution, in the posterior bladder wall, or alternatively, three to four injections of 2 mL each at the equatorial line of the bladder, with no apparent compromise in efficacy [11,12,13]. Investigations into injections in the trigone have been predicated on the premise that this region of the bladder harbors the highest density of sensory fibers. It is hypothesized that a combination of injections in the bladder walls and trigone may yield greater effectiveness than injections confined solely to the bladder walls in cases of OAB [14,15]. More recently, some studies explored further the reduced number of injections and a different location, offering reassurance that fewer injections seem equally effective, and that trigone inclusion could be as, or even more, effective, as targeting the lower bladder could reduce the urinary retention by maintaining upper bladder function [16,17,18]. The depth of injections, whether intradetrusor or suburothelial, has been the subject of a meta-analysis, which found no discernible differences in efficacy or safety. Large-scale clinical trials have not detected antibody formation following initial administration. However, to mitigate the risk of immunosensitization, a 12-week interval between injection cycles is recommended [9,10].

This treatment has successfully addressed a significant percentage of patients who did not respond to previous interventions, with effects lasting for an average of 6 months, according to pivotal trials [14,19]. The primary complications of intradetrusor injections of botulinum toxin A are urinary tract infections and urinary retention. Patients should be informed that they might require temporary (self-)catheterization. Yet, the treatment offers the advantage of virtually no systemic side effects [20].

As UI prevalence is high and its impact on women’s lives is substantial, it is imperative to conduct a comprehensive examination of all facets related to its treatment to ensure the delivery of increasingly efficacious care [21,22]. Notwithstanding this, probably because its efficacy has already been established, recent studies analyzing it are scarce, especially in an idiopathic context and even more specifically in the female population. This scarcity is even more pronounced if we consider it from a patient-reported-outcomes perspective, or with apparently less objective measures such as the number of pads, which may actually be the best surrogates for a patient’s quality of life. Furthermore, the latest evaluations available focus on urodynamic parameters. Notably, one of the key urodynamic markers for OAB is detrusor hyperactivity, which is not always necessary for diagnosis.

Therefore, when we say that the primary objective of this study is to assess the efficacy of botulinum toxin, we are addressing something directly applicable to real-life practice: this can inform a patient about the probability of no longer needing to use pads following the treatment. It is remarkable that 74.5% of patients reported a complete discontinuation of pad usage post treatment, but it is essential to acknowledge that this rate includes only those achieving complete dryness, as there were women still employing pads but who experienced significant improvements, leading to great changes in their quality of life. As such, of the remaining 25.5% patients, some exhibited a positive response to Botox; and some, with a negative response in the first treatment, underwent a second treatment to address dosage and technical issues as potential causes of the initial failure.

As the main determinants of efficacy, we identified the number of pads used before treatment and a history of sling placement, both exerting influence on treatment outcomes. The former is inherently predictable, given the association between the severity of UI and treatment challenges. Furthermore, certain patients not responsive to conservative management, due to antimuscarinic intolerance, may signify a more easily treatable condition, correlating with a higher success rate [23]. However, the latter finding deviates from prior reports demonstrating similar efficacy in women with or without a history of previous sling surgery [24]. In the present investigation, there appears to be a trend towards a less favorable clinical outcome in patients with a history of prior sling procedures. Despite timely reassessment for obstruction in patients upon UUI development, one could argue that sling surgery often augments bladder outlet resistance, yielding functional consequences even in cases with low post-void residual and high flow rates. Supporting this contention, urinary NGF, elevated in female rats with bladder outlet obstruction, was similarly elevated in reports of patients after sling placement, comparable to levels previously described in patients with overactive bladder [25]. Moreover, some studies suggest that increased bladder outlet resistance after a sling procedure may significantly contribute to the rise of urinary neurotrophins, promoting the sensitization of bladder primary afferents and inducing de novo urgency in susceptible patients [26]. This finding could prompt a new line of inquiry into whether patients with a sling truly have idiopathic UUI or if the sling itself was the cause. In both cases (high number of pads used before treatment and a history of sling placement), we should be more emphatic explaining to the patient the lower probability of success, as expectations are decisive in the therapeutic process. These will be the most challenging patients, but also the ones more in need for a solution.

Despite an average interval of approximately 6 months, pivotal trials indicate the need for repeat injections with intervals ranging from 3 months to over a year [27,28]. Patients received a second injection after a median duration of 18 months. Although surprisingly long, it is important to note that this interval does not necessarily equate to the duration of therapeutic effect. Over nearly 15 years, various factors contributed to this timeframe, albeit some no longer relevant today. Logistical challenges, including temporary follow-up losses and delayed post-Botox evaluations, contributed to this timeframe, with patients often waiting excessively to consult their urologist. Additionally, some patients were still pleased with the tail effect of the previous toxin injection, which means that, although lacking the full influence from the treatment, they were still satisfied, not seeking a new one. It is also noteworthy that some women opted to defer a second treatment even when the initial one was no longer effective, primarily due to the invasiveness of the procedure, carried out in the operating room under sedation, prompting a desire to avoid it.

This highlights the necessity of finding a novel method of botulinum toxin administration. To date, the administration requires the injection of a toxin solution in the bladder wall since the toxin does not traverse the urothelium if instilled in the bladder [29]. Various facilitators for enhancing the transurothelial passage of the toxin have been explored, including liposomes, the reverse thermal gelation TC-3 hydrogel, low-energy shock waves, and electromotive administration [30,31,32,33]. However, botulinum toxin encapsulated in liposomes did not produce results robust enough to pursue its development in OAB. It also failed when mixed in reverse thermal biodegradable hydrogel that increases viscosity at body temperature. Three thousand low-energy shock waves delivered during 10 min to the bladder half-filled with a solution containing 100U onabotA provided short-lasting effects. Electromotive administration has also been investigated without success. Facilitators for the passage of the toxin through the urothelium have so far failed or did not show irrefutable efficacy. The use of the needle in a flexible cystoscope, with a local anesthetic agent such as lidocaine, may also allow to perform an in-office procedure, which is simpler for the patients to repeat [34].

This phenomenon is interconnected with the repetition of the procedure across the years. While age at diagnosis and satisfaction emerged as determinants for procedure repetition, certain patients, even those young and satisfied with the results, opted to postpone it even when already losing efficacy. We attribute it to the aforementioned reasons.

Although older patients of the cohort appeared to request fewer treatments, the issue of age prompts further reflection. While the safety of onabotulinumtoxinA administration in elderly patients has been established, the same is not entirely true for previous treatment lines such as anticholinergics. In fact, the chronic use of anticholinergic drugs is increasingly being challenged due to growing evidence that prolonged exposure may lead to cognitive deterioration, particularly among elderly patients already at risk of dementia [35]. Moreover, a systematic review has shown that botulinum toxin injections are significantly more effective in curing UUI compared to any form of oral medication [36]. Given these considerations, should intravesical botulinum toxin be contemplated or even prioritized before anticholinergics, in an aging population?

Another matter of concern is the loss of efficacy over time. While subjective patient perceptions in clinical practice suggest diminishing efficacy with subsequent injections, our findings did not corroborate this observation. Furthermore, studies indicate a reduction in urgency severity associated with long-term therapeutic effects, aligning with real-world practice observations [37].

The principal limitations of our results stem from the retrospective nature of the study and the heterogeneity of clinical records among urologists, including the absence of standardized questionnaires or bladder diaries, which complicates the objective quantification of patient improvement without introducing biases. There are several points where this could be more evident. First, as the number of pads used is not consistently reported in clinical practice, the magnitude of improvement with the injections is probably higher than indicated. Conversely, complications from the treatment are likely underreported, as some are resolved before patients contact their urologist or are not detailed in clinical records. Additionally, demographics such as BMI, which we did not find to correlate with outcomes, might have shown correlations if we had a larger dataset, as analyses from randomized studies showed that a higher BMI is associated with a decreased time to recurrence [38]. Finally, regarding patient selection, it is possible that relevant information from patient clinical history was not recorded, preventing us from excluding women with identified causes of UUI.

As we acknowledge that many of the limitations of this study are due to its retrospective nature, future research should ideally be conducted prospectively, with all research questions defined in advance. Women for whom the treatment is effective but still decide to discontinue are intriguing. Does the procedural context—typically performed in an operating room—play a significant role? Do they never return to their baseline status and feel good enough? These are questions that remain to be addressed.

## 4. Conclusions

Intravesical botulinum toxin is a highly effective treatment. A clear correlation exists between a lower pre-procedural pad usage and a higher likelihood of achieving continence. Contrarily, patients with a history of sling placement for stress urinary incontinence who developed UUI exhibited a reduced likelihood of attaining continence. Furthermore, younger women at the time of diagnosis demonstrated an increased inclination to seek repeated injections, a trend mirrored in patients expressing greater satisfaction with the initial procedure. Surprisingly, in real-life conditions, patients often delayed the next injection longer than anticipated.

## 5. Materials and Methods

This is a retrospective observational cohort study. Women over 18 years old with a diagnosis of UUI, included or not in the OAB syndrome, were included if they were refractory to conservative treatment. All eligible patients had not been adequately managed with one or more anticholinergics and/or b3-adrenergic receptor agonists for UUI treatment, due to insufficient efficacy or intolerable side effects. Patients underwent treatment with botulinum toxin injection between 2010 and 2023. Women with an identified cause for their clinical condition were excluded, as well as those whose first treatment was performed in the last quarter of 2023, as it was deemed insufficient time for a proper reassessment of the need for new injections.

OnabotulinumtoxinA (Botox^®^, Allergan, Inc., Irvine, CA, USA) was the administered toxin in all patients. It was administered under light sedation using a rigid cystoscope and a 22-gauge needle. The product was injected at 20 sites, with each injection delivering 0.5 mL, distributed throughout the bladder [11]. In recent years, more injections were positioned below the equatorial line. To enhance the distribution area of onabotulinumtoxinA, the bladder was filled with 200 mL of saline. Prophylactic antibiotics were administered concurrently with sedation using a third-generation cephalosporin. Following the procedure, the patient was catheterized, and the catheter was removed a few hours later. However, in recent years, no bladder catheter was left in place post procedure, and the bladder was merely emptied.

Post-operatively, patients were assessed by different doctors, at various time intervals.

The primary outcome was efficacy, evaluated through the cessation of the need for pads. Secondary outcomes included the time between injections, determinants of efficacy, and patient satisfaction assessed through an affirmative response to the question “Are you satisfied?”. Variables such as age at diagnosis, BMI, prior suburethral sling placement before UUI diagnosis, the number of pads used before and after the intervention, the number of botulinum toxin treatments, the time between each treatment, and patient satisfaction were assessed. Complications were also measured.

The statistical analysis was conducted using IBM SPSS Statistics version 26.0 (IBM Corp., Armonk, NY, USA). A descriptive analysis of the sample was performed. Frequencies were expressed in percentages, and continuous variables were presented as means and medians, with their respective standard deviation and interquartile range. Student’s *t*-test and chi-square tests were used, considering a *p*-value < 0.05 as statistically significant. Binary logistic regression was used to evaluate the independent predictive value of the number of pre-injection pads and the previous use of a sling, adjusted for age and BMI, as predictors of the primary outcome.

This study was approved by the ethics committee of the Centro Hospitalar Universitário São João (CES 293/2023).

## Figures and Tables

**Table 1:** Number of patients by number of treatments requested after the first procedure.

Number of Treatments	Number of Patients (%)
0	146 (39.7)
1	114 (30.9)
2	61 (16.6)
3	24 (6.5)
4	8 (2.2)
5	7 (1.9)
6	6 (1.6)
7	1 (0.3)
8	1 (0.3)

## Data Availability

The original contributions presented in the study are included in the article, further inquiries can be directed to the corresponding author.
